# Addiction Transfer Post Bariatric Surgery- A Case Report

**DOI:** 10.1192/j.eurpsy.2024.1162

**Published:** 2024-08-27

**Authors:** S. Haugh, H. Belay, P. Cromwell

**Affiliations:** ^1^Department of Psychiatry, Connolly Hospital; ^2^National Bariatric Unit Ireland, St Vincents University Hospital, Dublin, Ireland

## Abstract

**Introduction:**

Bariatric surgery is an effective treatment for patients with obesity. Rates of obesity are increasing worldwide as are the number of bariatric procedures performed. Following bariatric surgery patients have increased contact with psychiatric services, there is an increased risk of deliberate self-harm, suicide attempts and completed suicide. Compared to the general population there is 8 fold higher than average suicide rate. In Ireland wait lists for bariatric surgery are long, resulting in many patients seeking surgery abroad. Bariatric ‘tourism’ often results in reduced psychological supports both pre and post op as well as reduced pre surgical screening for psychiatric illness. Bariatric surgery is also associated with ‘addiction transfer’. The literature suggests that patients often substitute the maladaptive coping mechanism of eating with other impulsive behaviors such as substance misuse or gambling.

**Objectives:**

Case report highlighting the issue of addiction transfer among patients that have undergone bariatric surgery.

**Methods:**

Case report: A 38 year old woman admitted to the acute psychiatric unit with self harm, suicidal ideation, low mood, and recent overdose of venlafaxine. On initial presentation, she was intoxicated with alcohol, her toxicology was positive for cocaine and benzodiazepines. She had undergone a gastric bypass 14 months previous, having travelled abroad to have the procedure. She had not attended for any bariatric follow up with her GP post operatively. She was not taking any vitamins post operatively despite advice from the clinic. The patient was admitted to the acute psychiatric unit. She admitted to drinking excessively in the last year. She denied any history of mood disturbance or substance or alcohol misuse prior to surgery. She had no previous contacts with psychiatric services. Her GP had commenced her on venlafaxine for low mood 6 months prior to psychiatric admission. She was admitted to the acute unit for 5 days after which she left against medical advice. She was followed up in the day hospital and referred to addiction services.

**Results:**

case report

**Conclusions:**

There is growing evidence about the psychiatric and addiction implications of bariatric surgery. Offering psychological support for patients post operatively is essential. Unfortunately, because of long wait lists in Ireland many patients chose to travel abroad and often are unable to avail of MDT support. The emerging field of bariatric psychiatry could provide a useful addition to the bariatric specialist services.

**Disclosure of Interest:**

None Declared

